# Supplementary material for: Systematic Identification of the Optimal Housekeeping Genes for Accurate Transcriptomic and Proteomic Profiling of Tissues following Complex Traumatic Injury
Source: Methods Protoc. 2023 Feb 23;6(2):22. doi: 10.3390/mps6020022 (PMC10037587; doi:10.3390/mps6020022)
Supplement: Supplementary file 1 [file mps-06-00022-s001.zip › mps-2142213-supplementary.pdf]

methods and protocols

MDPI

Supplementary Materials

Table S1. List of common reference housekeeping genes based on a targeted literature search conducted using the PubMed database (2013-2023) involving qPCR data normalization studies across diverse spectrums of tissues, disease models, and animal species. Values indicate the number of peer-reviewed articles that mentioned or used a particular HKG in the context of qPCR data normalization.

	"Housekeeping gene"	"Reference"	"Internal control"	"qPCR reference"
Gapdh	513	1053	398	499
Actb	202	433	81	256
Tbp	140	311	54	139
B2m	132	277	59	161
Hprt1	101	206	33	123
Pgk1	50	77	14	41
Rplp0	40	70	12	43
Tfrc	35	49	8	23
Rplp2	2	7	0	4

Table S2. List and characteristics of antibodies used in this study.

Antibody	Brand	Catalog number	Dilution used
ACTB	Abcam	Ab49900	1:30,000
B2M	Novus Biologicals	NBP2-92971	1:500
GAPDH	Cell Signaling	5174	1:5,000
HPRT1	Novus Biologicals	NBP1-92006	1:1,000
PGK1	Santa Cruz	SC-130335	1:3,000
RPLP0	Abcam	AB192866	1:800
RPLP2	Novus Biologicals	NBP2-33688	1:800
TBP	Cell Signaling	8515S	1:800
TFRC	Novus Biologicals	NB100-92243SS	1:800

Table S3. Calculated mean of Ct values, standard deviations and coefficients of gene expression variation (CV) for the 9 housekeeping genes under steady state and post- injury in lung, liver, kidney and skeletal muscle (n≥ 4 animals/group).

Lung	Naïve	6 h Post-Injury	24 h Post-Injury	168 h Post-Injury	All Injury time-points	Naïve+ All time-points
Actb	23.77 ± 2.22<br>*CV: 0.093*	29.87 ± 3.97<br>*CV: 0.133*	24.54 ± 1.13<br>*CV: 0.046*	24.64 ± 0.81<br>*CV: 0.033*	26.25 ± 3.40<br>*CV: 0.130*	25.09 ± 3.16<br>*CV: 0.126*
B2m	20.50 ± 1.87<br>*CV: 0.091*	25.03 ± 2.66<br>*CV: 0.106*	20.77 ± 1.59<br>*CV: 0.077*	20.86 ± 1.04<br>*CV: 0.050*	21.94 ± 2.58<br>*CV: 0.118*	21.25 ± 2.38<br>*CV: 0.112*
Gapdh	24.46 ± 1.42<br>*CV: 0.058*	26.67 ± 1.38<br>*CV: 0.052*	23.65 ± 1.63<br>*CV: 0.069*	24.76 ± 0.76<br>*CV: 0.031*	24.90 ± 1.73<br>*CV: 0.069*	24.69 ± 1.60<br>*CV: 0.065*
Hprt1	25.08 ± 1.42<br>*CV: 0.057*	27.41 ± 1.93<br>*CV: 0.070*	24.18 ± 0.86<br>*CV: 0.036*	24.64 ± 0.78<br>*CV: 0.032*	25.27 ± 1.85<br>*CV: 0.073*	25.17 ± 1.65<br>*CV: 0.066*
Pgk1	21.64 ± 0.55<br>*CV: 0.025*	22.22 ± 1.80<br>*CV: 0.081*	20.89 ± 0.46<br>*CV: 0.022*	21.70 ± 0.33<br>*CV: 0.015*	21.61 ± 1.19<br>*CV: 0.055*	21.62 ± 0.96<br>*CV: 0.044*

Rplp0	19.38 ±1.12<br>*CV: 0.058*	22.3 ± 2.82<br>*CV: 0.126*	18.87 ± 0.74<br>*CV: 0.039*	19.18 ± 0.49<br>*CV: 0.026*	20.06 ± 2.25<br>*CV: 0.112*	19.74 ± 1.85<br>*CV: 0.094*
Rplp2	22.61 ± 1.63<br>*CV: 0.072*	26.83 ± 3.10<br>*CV: 0.116*	22.33 ± 1.02<br>*CV: 0.046*	22.55 ± 0.64<br>*CV: 0.028*	23.82 ± 2.76<br>*CV: 0.116*	23.26 ± 2.38<br>*CV: 0.102*
Tbp	27.71 ± 2.17<br>*CV: 0.078*	31.58 ± 2.35<br>*CV: 0.074*	27.78 ± 0.92<br>*CV: 0.033*	28.58 ± 0.76<br>*CV: 0.027*	29.11 ± 2.08<br>*CV: 0.071*	28.44 ± 2.23<br>*CV: 0.078*
Tfrc	28.13 ± 1.55<br>*CV: 0.055*	30.06 ± 0.96<br>*CV: 0.032*	28.03 ± 1.01<br>*CV: 0.036*	29.08 ± 0.49<br>*CV: 0.017*	28.99 ± 1.14<br>*CV: 0.039*	28.59 ± 1.41<br>*CV: 0.049*
**Liver**	**Naïve**	**6 h Post-Injury**	**24 h Post-Injury**	**168 h Post-Injury**	**All Injury time-points**	**Naïve+ All time-points**
Actb	25.44 ± 1.31<br>*CV: 0.051*	29.92 ± 5.08<br>*CV: 0.170*	29.12 ± 3.95<br>*CV: 0.136*	30.75 ± 5.11<br>*CV: 0.166*	29.93 ± 4.79<br>*CV: 0.160*	28.05 ± 4.35<br>*CV: 0.155*
B2m	20.59 ± 1.25<br>*CV: 0.061*	23.71 ± 2.94<br>*CV: 0.124*	23.28 ± 1.94<br>*CV: 0.083*	24.84 ± 3.34<br>*CV: 0.134*	23.94 ± 2.88<br>*CV: 0.120*	22.54 ± 2.85<br>*CV: 0.126*
Gapdh	22.86 ± 1.41<br>*CV: 0.062*	22.56 ± 1.10<br>*CV: 0.049*	22.92 ± 0.73<br>*CV: 0.032*	25.33 ± 1.34<br>*CV: 0.053*	23.61 ± 1.64<br>*CV: 0.069*	23.29 ± 1.59<br>*CV: 0.068*
Hprt1	25.55 ± 1.07<br>*CV: 0.042*	25.14 ± 1.02<br>*CV: 0.041*	25.44 ± 0.32<br>*CV: 0.013*	28.93 ± 2.84<br>*CV: 0.098*	26.50 ± 2.46<br>*CV: 0.093*	26.10 ± 2.05<br>*CV: 0.079*
Pgk1	21.91± 0.51<br>*CV: 0.023*	21.28 ± 0.75<br>*CV: 0.035*	20.83 ± 0.31<br>*CV: 0.015*	24.66 ± 3.11<br>*CV: 0.126*	22.26 ± 2.52<br>*CV: 0.113*	22.11 ± 1.96<br>*CV: 0.089*
Rplp0	20.90 ± 0.58<br>*CV: 0.028*	18.58 ± 0.78<br>*CV: 0.042*	18.32 ± 0.38<br>*CV: 0.021*	23.03 ± 2.72<br>*CV: 0.118*	19.98 ± 2.71<br>*CV: 0.136*	20.37 ± 2.15<br>*CV: 0.106*
Rplp2	23.50 ± 1.00<br>*CV: 0.043*	21.43 ± 0.86<br>*CV: 0.040*	20.88 ± 0.94<br>*CV: 0.045*	25.75 ± 1.81<br>*CV: 0.070*	22.68 ± 2.52<br>*CV: 0.111*	23.03 ± 2.07<br>*CV: 0.090*
Tbp	29.25 ± 1.11<br>*CV: 0.038*	27.21 ± 0.67<br>*CV: 0.025*	27.51 ± 0.67<br>*CV: 0.024*	34.61 ± 4.50<br>*CV: 0.130*	29.78 ± 4.33<br>*CV: 0.145*	29.56 ± 3.38<br>*CV: 0.114*
Tfrc	27.86 ± 1.35<br>*CV: 0.048*	26.98 ± 0.85<br>*CV: 0.032*	25.84 ± 0.78<br>*CV: 0.030*	32.54 ± 4.18<br>*CV: 0.128*	28.46 ± 3.85<br>*CV: 0.135*	28.21 ± 3.07<br>*CV: 0.109*
**Kidney**	**Naïve**	**6 h Post-Injury**	**24 h Post-Injury**	**168 h Post-Injury**	**All Injury time-points**	**Naïve+ All time-points**
Actb	25.96 ± 1.47<br>*CV: 0.057*	25.33 ± 1.02<br>*CV: 0.040*	29.51 ± 7.47<br>*CV: 0.253*	25.95 ± 0.93<br>*CV: 0.036*	26.93 ± 4.75<br>*CV: 0.176*	26.52 ± 3.78<br>*CV: 0.143*
B2m	24.07 ± 1.35<br>*CV: 0.056*	23.49 ± 1.33<br>*CV: 0.057*	23.25 ± 3.50<br>*CV: 0.151*	23.67 ± 0.48<br>*CV: 0.020*	23.47 ± 2.19<br>*CV: 0.093*	23.72 ±1.90<br>*CV: 0.080*
Gapdh	23.38 ± 1.15<br>*CV: 0.049*	22.71 ± 0.73<br>*CV: 0.032*	21.72 ± 0.86<br>*CV: 0.040*	24.10 ± 0.69<br>*CV: 0.029*	22.84 ± 1.24<br>*CV: 0.054*	23.06 ±1.24<br>*CV: 0.054*
Hprt1	27.32 ± 0.94<br>*CV: 0.034*	25.70 ± 0.79<br>*CV: 0.031*	24.74 ± 0.72<br>*CV: 0.029*	27.82 ± 0.72<br>*CV: 0.026*	26.09 ± 1.48<br>*CV: 0.057*	26.60 ±1.42<br>*CV: 0.053*
Pgk1	22.29 ± 0.56<br>*CV: 0.025*	20.52 ± 0.66<br>*CV: 0.032*	19.9 5 ± 0.31<br>*CV: 0.016*	22.60 ± 0.51<br>*CV: 0.023*	21.03 ± 1.25<br>*CV: 0.059*	21.56 ±1.20<br>*CV: 0.056*
Rplp0	21.34 ± 0.58<br>*CV: 0.027*	19.43 ± 0.66<br>*CV: 0.034*	18.63 ± 0.36<br>*CV: 0.019*	20.98 ± 0.46<br>*CV: 0.022*	19.68 ± 1.10<br>*CV: 0.056*	20.38 ±1.23<br>*CV: 0.060*
Rplp2	23.76 ± 0.74<br>*CV: 0.031*	22.22 ± 0.69<br>*CV: 0.031*	21.52 ± 0.59<br>*CV: 0.027*	23.95 ± 0.69<br>*CV: 0.029*	22.57 ± 1.22<br>*CV: 0.054*	23.07 ±1.20<br>*CV: 0.052*
Tbp	30.26 ± 1.20<br>*CV: 0.040*	28.25 ± 0.65<br>*CV: 0.023*	27.82 ± 0.34<br>*CV: 0.012*	30.69 ± 0.45<br>*CV: 0.015*	28.92 ± 1.36<br>*CV: 0.047*	29.45 ±1.45<br>*CV: 0.049*
Tfrc	28.63 ± 1.19<br>*CV: 0.042*	26.92 ± 0.83<br>*CV: 0.031*	26.25 ± 0.27<br>*CV: 0.010*	29.67 ± 0.70<br>*CV: 0.024*	27.61 ± 1.61<br>*CV: 0.058*	28.04 ±1.54<br>*CV: 0.055*
**Muscle**	**Naïve**	**6 h Post-Injury**	**24 h Post-Injury**	**168 h Post-Injury**	**All Injury time-points**	**Naïve+ All time-points**
Actb	28.85 ± 2.93<br>*CV: 0.102*	26.42 ± 1.83<br>*CV: 0.069*	26.10 ± 1.31<br>*CV: 0.050*	23.86 ± 1.06<br>*CV: 0.044*	25.46 ± 1.83<br>*CV: 0.072*	26.67 ± 2.80<br>*CV: 0.105*
B2m	24.77 ± 3.10<br>*CV: 0.125*	22.35 ± 1.73<br>*CV: 0.077*	22.08 ± 1.27<br>*CV: 0.058*	19.72 ± 0.99<br>*CV: 0.050*	21.38 ± 1.81<br>*CV: 0.085*	22.59 ± 2.86<br>*CV: 0.127*
Gapdh	21.38 ± 3.73<br>*CV: 0.174*	19.47 ± 2.65<br>*CV: 0.136*	19.20 ±1.29<br>*CV: 0.067*	18.94 ± 1.08<br>*CV: 0.057*	19.20 ± 1.83<br>*CV: 0.095*	19.98 ± 2.86<br>*CV: 0.143*
Hprt1	26.95 ± 3.40	25.13 ± 2.50	23.93 ± 1.52	23.39 ± 0.73	24.15 ± 1.89	25.15 ± 2.87

	CV: 0.126	*CV: 0.099*	*CV: 0.064*	*CV: 0.031*	*CV: 0.078*	*CV: 0.114*
Pgk1	19.18 ± 1.17<br>*CV: 0.061*	19.39 ± 1.81<br>*CV: 0.093*	18.97 ± 0.50<br>*CV: 0.026*	20.11 ± 0.80<br>*CV: 0.040*	19.49 ± 1.27<br>*CV: 0.065*	19.38 ± 1.24<br>*CV: 0.064*
Rplp0	21.15 ± 2.34<br>*CV: 0.111*	20.48 ± 1.85<br>*CV: 0.090*	18.82 ± 0.80<br>*CV: 0.043*	18.54 ± 0.50<br>*CV: 0.027*	19.28 ± 1.47<br>*CV: 0.076*	19.95 ± 2.04<br>*CV: 0.102*
Rplp2	24.64 ± 2.89<br>*CV: 0.117*	22.78 ± 1.98<br>*CV: 0.087*	22.09 ± 1.33<br>*CV: 0.060*	20.90 ± 0.55<br>*CV: 0.026*	21.92 ± 1.61<br>*CV: 0.073*	22.89 ± 2.52<br>*CV: 0.110*
Tbp	30.92 ± 3.27<br>*CV: 0.106*	29.49 ± 2.53<br>*CV: 0.086*	28.62 ± 1.37<br>*CV: 0.048*	27.36 ± 0.56<br>*CV: 0.020*	28.49 ± 1.91<br>*CV: 0.067*	29.36 ± 2.74<br>*CV: 0.093*
Tfrc	27.01 ± 3.42<br>*CV: 0.127*	27.29 ± 2.02<br>*CV: 0.074*	28.71 ± 1.02<br>*CV: 0.036*	26.35 ± 0.88<br>*CV: 0.033*	27.45 ± 1.70<br>*CV: 0.062*	27.29 ± 2.47<br>*CV: 0.091*

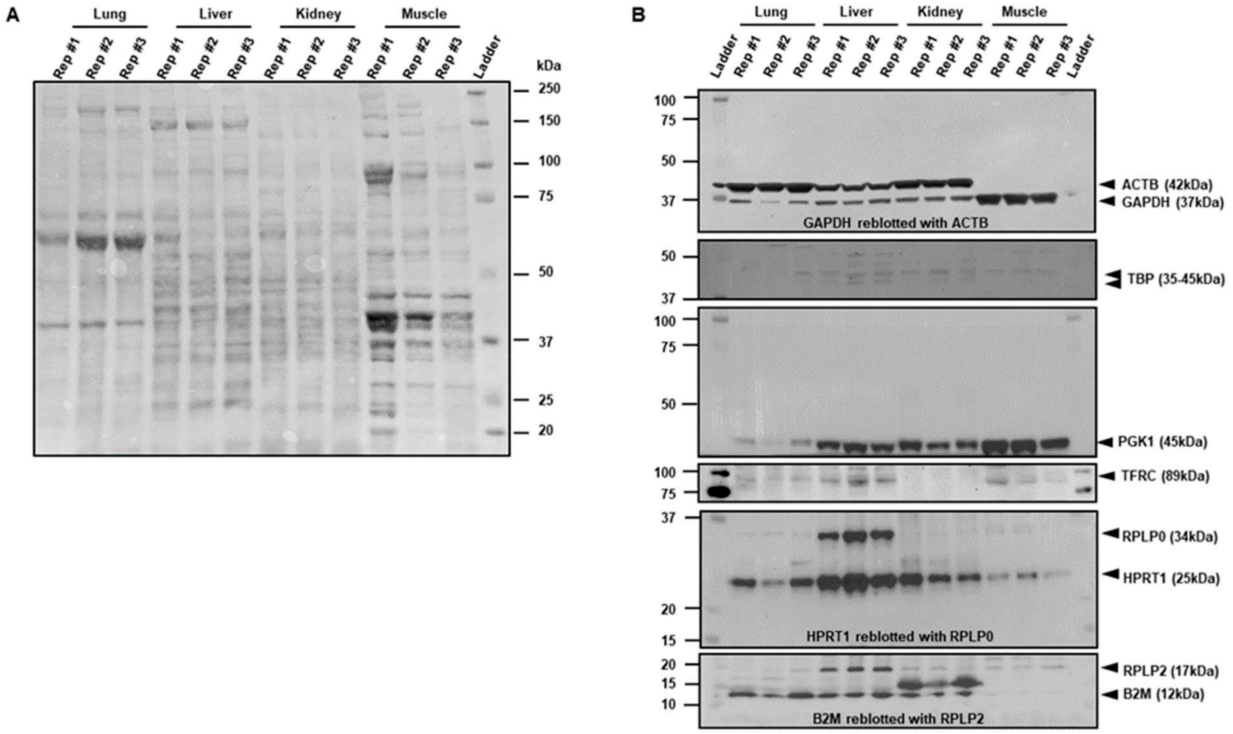

Figure S1. Representative Ponceau S and Western blot images. **(A)** Representative Ponceau S total protein staining following protein transfer from SDS-Page gels to nitrocellulose membranes. **(B)** Immunoblots against 9 HKGs for naïve lung, liver, kidney and muscle tissue (15 µg of total protein loaded lane; n = 3 biological replicates indicated as #1, #2 and #3).

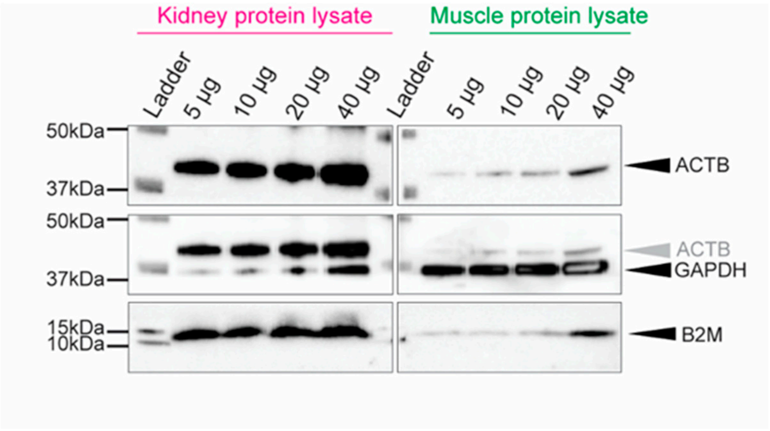

Figure S2. Immunoblots against ACTB, GAPDH and B2M using different concentrations of total protein from kidney and skeletal muscle tissue lysates. Note: ACTB membrane was re-probed for GAPDH.

LUNG

Genes	Geomean of ranking values NAÏVE+INJURED
Rplp2/Tfrc/Rplp0	1.41
Rplp2/Rplp0	1.68
Rplp0/Tfrc	3
Rplp0	3.34
Rplp2/Tfrc	4.43
Rplp2	6.24
Tfrc	6.44

LIVER

Genes	Geomean of ranking values NAÏVE+INJURED
Hprt1/Pgk1/Tbp	1.41
Pgk1/Tbp	2.11
Hprt1/Pgk1	2.91
Pgk1	3.83
Hprt1/Tbp	4.12
Hprt1	4.40
Tbp	7

KIDNEY

Genes	Geomean of ranking values NAÏVE+INJURED
Pgk1/Rplp0/Rplp2	1.41
Pgk1/Rplp2	1.68
Rplp0/Rplp2	3
Rplp2	4.3
Pgk1/Rplp0	4.43
Rplp0	5.44
Pgk1	5.73

MUSCLE

Genes	Geomean of ranking values NAÏVE+INJURED
Rplp0/Rplp2/Tbp	1.41
Rplp0/Tbp	1.86
Rplp0/Rplp2	2.71
Rplp0	4.3
Rplp2	4.47
Rplp2/Tbp	4.95
Tbp	6.24

Most stable — Least stable

Figure S3. Gene stability ranking obtained with RefFinder using the three most stable HKG by comparing either single HKG or HKG combinations of two or three genes. The color-scale represents the most (yellow) to least (blue) stable HKG.
